# Risk factors for peripancreatic and pancreatic infection of acute pancreatitis and the development of a clinical prediction model

**DOI:** 10.1097/MD.0000000000042595

**Published:** 2025-05-23

**Authors:** Yu Zhang, Shuaiyong Wen, Guijie Zhao, Yunfeng Cui

**Affiliations:** a Tianjin NanKai Hospital, Tianjin Medical University, Tianjin, China; b Tianjin Key Laboratory of Acute Abdomen Disease Associated Organ Injury and ITCWM Repair, Tianjin, China; c Institute of Integrative Medicine for Acute Abdominal Diseases, Tianjin, China; d Department of Hepatobiliary and Pancreatic Surgery, Department of Surgery, Tianjin Nankai Hospital, Nankai Clinical School of Medicine, Tianjin Medical University, Tianjin, China.

**Keywords:** abdominal infection, acute pancreatitis, clinical prediction model, infected necrotizing pancreatitis, predicting, risk factors

## Abstract

Infected necrotizing pancreatitis (INP) is a serious complication that can increase the length of hospital stay and the cost of treatment, and is the leading cause of death in patients with acute pancreatitis (AP). This article aimed to predict the possibility of pancreatic and peripancreatic infections by early clinical indicators of AP and construct a clinical prediction model. We retrospectively studied consecutive patients admitted to the Nankai Hospital for moderate severe AP and severe AP, which developed within 2 weeks. Logistic regression was used to evaluate potential factors that could lead to INP and to develop clinical prediction model. Persistent organ failure, pancreatic necrosis area, and procalcitonin account were risk factors for INP. A prediction model was constructed based on the risk factors. The results showed that the model had good predictive performance. We developed a clinical prediction model with good predictive results that can be helpful for clinicians to identify and prevent the development of INP at an early stage.

## 1. Introduction

Acute pancreatitis (AP) is a common acute abdominal disease that includes mild acute pancreatitis (MAP), moderate severe acute pancreatitis (MSAP), and severe acute pancreatitis (SAP) according to the severity of the Atlanta Classification.^[1]^ Approximately 80% of patients have MAP that is self-limiting. However, necrotizing pancreatitis occurs in 15% to 20% of patients with AP, 30% of whom develop infected necrotizing pancreatitis (INP), resulting in high mortality rates, prolonged hospital stays and significant spending.^[2,3]^ Pancreatic necrosis is one of the complications of AP and is thought to be related to the destruction of the main pancreatic duct within the region of parenchymal necrosis. The intestinal damage caused by AP increases the permeability of the intestinal mucosa and damages intestinal microorganisms, leading to microbial infection of necrotic pancreatic tissue through the blood, lymph nodes or gastrointestinal tract.^[1,3,4]^ Despite major advances in the treatment of INP, the mortality rate remains high. Therefore, the prevention and management of INP remain clinical problems that need to be addressed.

Multiple studies have found that some laboratory markers and scoring systems can predict the severity of AP, but clinical utility remains limited.^[5]^ The main objective of this study was to analyze clinical indicators in MSAP and SAP patients in the early stages of onset (<2 weeks) to investigate risk factors for secondary INP, to develop a clinical prediction model, and to provide an overview of the treatment of INP and the clinical outcome of patients at our center.

## 2. Methods

### 2.1. Study design and patient collection

**Figure 1.**
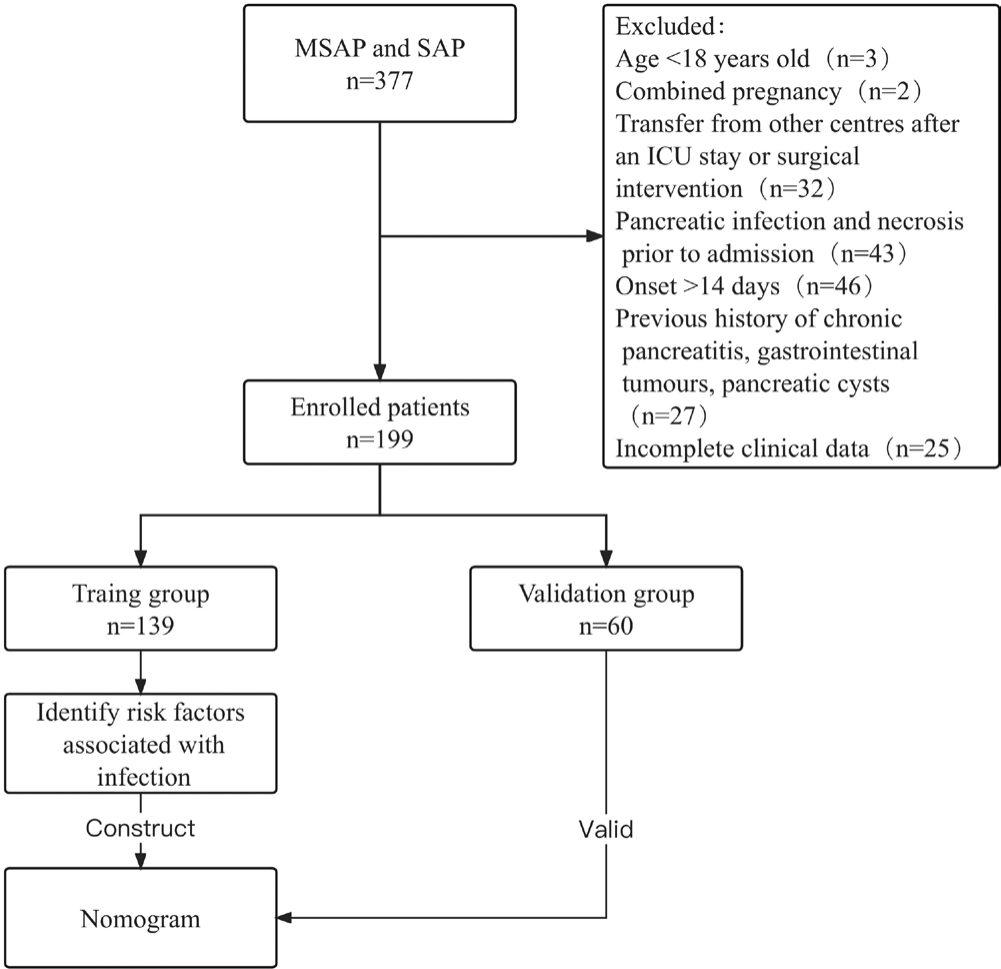
Flow chart of patients admitted to our center with MSAP and SAP. ICU = intensive care unit, MSAP = moderate severe acute pancreatitis, SAP = severe acute pancreatitis.

We retrospectively collected data from patients who were consecutively admitted with a diagnosis of MSAP or SAP with an onset of <2 weeks to the Department of Hepatobiliary and Pancreatic Surgery I, Nankai Hospital, from January 2019 to December 2022. The inclusion and exclusion criteria for patients are presented in Figure 1 in a flow chart.

### 2.2. Definition

We defined some of the clinical events and clinical indicators that were included in the study.

(1) According to the modified Atlantic Classification criteria,^[1]^ the diagnosis of AP is based on any 2 or more of the following 3 criteria: (1) abdominal pain consistent with the clinical manifestations of AP; (2) serum amylase and/or lipase activity at least 3 times above the normal upper limit; (3) imaging changes compatible with AP. SAP and SAP were defined as AP with transient and persistent organ failure (POF), respectively, according to the modified Marshall score.
(2) The presence of infection can be presumed when there is extraluminal gas in the pancreatic and/or peripancreatic tissues on contrast-enhanced computed tomography or when pancreatic and/or peripancreatic necrotic tissue obtained after drainage/surgery is positive according to the bacterial culture results.
(3) In this study, organ function was included in the statistical analysis as a risk factor for pancreatic infection. For the purpose of meeting the uniform criteria, organ failure (OF) was assessed individually using the Sequential Organ Failure Assessment (SOFA) score, and organ dysfunction was considered to be present if the SOFA score was ≥ 2. The definition of multiple organ dysfunction syndrome is a SOFA score of ≥ 2 in multiple organs. POF is defined as OF lasting ≥ 48 hours. Sepsis was defined as infection with SOFA score ≥ 2.^[6]^
(4) Acute compartment syndrome was defined as persistent intra-abdominal pressure (IAP) > 20 mm Hg combined with new OF.^[7]^

### 2.3. Data collection

The following data were collected from the enrolled patients: (1) demographic information (including age, underlying disease, history of smoking and drinking, etiological distribution, and body mass index); (2) assessment of pancreatitis and systemic function (including the Modified Computed Tomography Severity Index score and acute physiology and chronic health evaluation II [APACHE II score]); (3) organ function; (4) systemic complications; and (5) indicators of infection (including white blood cell, C-reactive protein, PCT, body fluid and blood culture results).

### 2.4. Statistics

Normally distributed continuous variables are expressed as the mean ± standard deviation (mean ± SD), and comparisons between groups were carried out by *t* tests; nonnormally distributed continuous variables are expressed as medians (interquartile ranges), and comparisons between groups were carried out by the Mann–Whitney *U* test. Categorical variables are expressed as numbers (percentages), and the chi-square test or Fisher exact test was applied. *P* < .05 suggested that the difference was statistically significant. The samples were randomly split into a training set and a validation set. After the inclusion indicators were subjected to univariate analysis, the statistically significant indicators were screened for logistic multivariate stepwise regression to establish a clinical prediction model, and R was used to construct a nomogram and plot the receiver operating characteristic (ROC) curve. The accuracy of the model was verified using calibration curves and the Hosmer–Lemeshow test. The clinical utility of the model was assessed by plotting DCA and internally validated by 1000 replicate samples using the bootstrap method. All the statistical analyses were performed with SPSS 26.0 and R 4.3.2.

## 3. Results

Of the 199 patients in the cohort, 88 progressed to INP. Table 1 shows the baseline characteristics of patients in the infected and non-infected groups. Among them, the APACHE II score and Modified Computed Tomography Severity Index score were greater (*P* ＜ .05) in infected patients than in uninfected patients. All patients were subsequently separated into a training group (n = 139) and a validation group (n = 60) according to a 7:3 random split. Table 2 describes the baseline characteristics of the patients in the training and difference groups, with no significant difference in the variability between the 2 groups.

**Table 1:** Baseline characteristics and other comorbidities in patients with and without infection.

	Total N = 199	Non-infected N = 111	Infected N = 88	*P*-value
Baseline characteristics				
Age (year)	41.00 (33.00–55.50)	40.00 (33.00–54.50)	41.50 (33.75–56.00)	.735
Males, n (%)	142 (71.36)	80 (72.07)	62 (70.45)	.802
BMI (kg/m^2^)	27.03 ± 4.17	27.18 ± 4.33	26.85 ± 3.97	.574
Cause of pancreatitis, n (%)				.356
Biliary	73 (36.68)	42 (37.84)	31 (35.23)	
Alcohol abuse	27 (13.57)	15 (13.51)	12 (13.64)	
Dyslipidaemia	72 (36.18)	39 (35.14)	33 (37.50)	
Post-ERCP	3 (1.51)	0 (0.00)	3 (3.41)	
Idiopathic	24 (12.06)	15 (13.51)	9 (10.23)	
Comorbidities, n (%)				
Hypertension	51 (25.63)	24 (21.62)	27 (30.68)	.146
Diabetes	44 (22.11)	26 (23.42)	18 (20.45)	.616
Hyperlipidemia	92 (46.23)	52 (46.85)	40 (45.45)	.845
Smoking	93 (46.73)	61 (54.95)	32 (36.36)	**.009**
Alcohol use	82 (41.21)	52 (46.85)	30 (34.09)	.069
APACHE II score	6.73 ± 4.80	5.66 ± 3.74	8.08 ± 5.61	**<.001**
MCTSI score	6.61 ± 1.55	6.32 ± 1.39	6.98 ± 1.66	**.003**

Bold values indicate statistical significance (*P* < .05).

APACHE II score = acute physiology and chronic health evaluation II, BMI = body mass index, MCTSI = Modified Computed Tomography Severity Index.

**Table 2:** Comparison of differences between the training group and validation group.

	Total N = 199	Train group N = 139	Valid group N = 60	*P*-value
Baseline characteristics				
Age (yr)	41.00 (33.00–55.50)	40.00 (33.00–54.50)	41.00 (34.00–57.00)	.399
Males, n (%)	142 (71.36)	102 (73.38)	40 (66.67)	.336
BMI (kg/m^2^)	27.03 ± 4.17	26.91 ± 4.37	27.32 ± 3.69	.532
Cause of pancreatitis, n (%)				.185
Biliary	73 (36.68)	48 (34.53)	25 (41.67)	
Alcohol abuse	27 (13.57)	18 (12.95)	9 (15.00)	
Dyslipidaemia	72 (36.18)	51 (36.69)	21 (35.00)	
Post-ERCP	3 (1.51)	1 (0.72)	2 (3.33)	
Idiopathic	24 (12.06)	21 (15.11)	3 (5.00)	
Comorbidities, n (%)				
Hypertension	51 (25.63)	33 (23.74)	18 (30.00)	.353
Diabetes	44 (22.11)	33 (23.74)	11 (18.33)	.399
Hyperlipidemia	92 (46.23)	65 (46.76)	27 (45.00)	.819
Smoking	93 (46.73)	70 (50.36)	23 (38.33)	.119
Alcohol use	82 (41.21)	59 (42.45)	23 (38.33)	.589
Pancreatic necrosis area, n (%)				.712
Non	85 (42.71)	58 (41.73)	27 (45.00)	
<30%	57 (28.64)	38 (27.34)	19 (31.67)	
30–50%	23 (11.56)	18 (12.95)	5 (8.33)	
>50%	34 (17.09)	25 (17.99)	9 (15.00)	
Complications, n (%)				
SIRS	49 (24.62)	37 (26.62)	12 (20.00)	.320
ARDS	35 (17.59)	25 (17.99)	10 (16.67)	.823
ACS	7 (3.52)	4 (2.88)	3 (5.00)	.744
Shock	27 (13.57)	19 (13.67)	8 (13.33)	.949
Sepsis	17 (8.54)	12 (8.63)	5 (8.33)	.945
OF	61 (30.65)	42 (30.22)	19 (31.67)	.839
MOF	32 (16.08)	23 (16.55)	9 (15.00)	.785
POF	40 (20.1)	28 (20.14)	12 (20.00)	.981
Type of OF, n (%)				
Respiratory failure	35 (17.59)	26 (18.71)	9 (15.00)	.529
Cardiovascular failure	21 (10.55)	16 (11.51)	5 (8.33)	.503
Renal failure	26 (13.07)	16 (11.51)	10 (16.67)	.322
Liver failure	16 (8.04)	10 (7.19)	6 (10.00)	.701
Gastrointestinal failure	14 (7.04)	7 (5.04)	7 (11.67)	.169
Coagulation failure	2 (1.01)	1 (0.72)	1 (1.67)	.513
Extrapancreatic infection, n (%)	30 (15.08)	19 (13.67)	11 (18.33)	.399
ICU stay, n(%)	55 (27.64)	38 (27.34)	17 (28.33)	.885
APACHE II score	6.73 ± 4.80	6.71 ± 4.88	6.78 ± 4.65	.916
MCTSI score	6.61 ± 1.55	6.62 ± 1.59	6.60 ± 1.44	.938
WBC* (×10^9^/L)	13.14 ± 4.85	13.07 ± 4.83	13.32 ± 4.92	.742
CRP* (mg/L)	172.94 (56.80–275.37)	161.01 (56.80–263.68)	193.40 (67.21–298.62)	.458
PCT* (mg/dL)	0.74 (0.23–2.92)	0.76 (0.24–3.22)	0.56 (0.18–1.88)	.251

ACS = abdominal compartment syndrome, APACHE II score = acute physiology and chronic health evaluation II, ARDS = acute respiratory distress syndrome, BMI = body mass index, CRP = C-reactive protein, ICU = intensive care unit, MCTSI = Modified Computed Tomography Severity Index, MOF = multiple organ failure, organ failure ≥2 was defined as MOF, OF = organ failure, PCT = procalcitonin, POF = persistent organ failure, organ failure ≥48 hours was defined as POF, SIRS = systemic inflammatory response syndrome, WBC = white blood cell.

* Consecutive maximum data within 3 days of admission.

The results of the univariate analysis are shown in Table 3. Among them, pancreatic necrosis area > 50% (*P* = .004), systemic inflammatory response syndrome (*P* = .029), acute respiratory distress syndrome (*P* = .002), shock (*P* = .007), OF (*P* < .001), multiple organ failure (*P* < .001), POF (*P* < .001), respiratory failure (*P* = .003), cardiovascular failure (*P* = .009), renal failure (*P* = .004), extrapancreatic infection (*P* = .008), intensive care unit stay (*P* < .001), APACHE II score (*P* = .004), white blood cell account (*P* = .018), C-reactive protein account (*P* = .030), and PCT account (*P* = .006) were statistically significant.

**Table 3:** Univariate analysis of patient baseline characteristics, complications and infectious indicators in uninfected and infected patients.

	Total N = 139	Non-infected N = 81	Infected N = 58	OR (95% CI)	*P*-value
Comorbidities, n (%)					
Hypertension	33 (23.74)	18 (12.95)	15 (10.79)	2.03 (0.93–4.40)	.074
Diabetes	33 (23.74)	19 (13.67)	14 (10.07)	0.64 (0.29–1.40)	.263
Hyperlipidemia	65 (46.76)	39 (28.06)	26 (18.71)	0.96 (0.49–1.87)	.910
Smoking	70 (50.36)	45 (32.37)	25 (17.99)	2.00 (1.01–3.96)	.046
Alcohol use	59 (42.45)	45 (32.37)	21 (15.11)	1.45 (0.73–2.88)	.292
Pancreatic necrosis, n (%)					
Non	58 (41.73)	39 (28.06)	19 (13.67)	1.00 (Reference)	
<30%	38 (27.34)	27 (19.42)	11 (7.91)	0.90 (0.40–2.02)	.791
30–50%	18 (12.95)	8 (5.76)	10 (7.19)	1.57 (0.53–4.65)	.411
>50%	25 (17.99)	7 (5.04)	18 (12.95)	5.95 (1.78–19.83)	**.004**
Complications, n (%)					
SIRS	37 (26.62)	17 (12.23)	20 (14.39)	2.44 (1.10–5.45)	**.029**
ARDS	25 (17.99)	7 (5.04)	18 (12.95)	6.24 (1.99–19.60)	**.002**
ACS	4 (2.88)	1 (0.72)	3 (2.16)	3.33 (0.34–32.81)	.303
Shock	19 (13.67)	6 (4.32)	13 (9.35)	4.90 (1.54–15.65)	**.007**
Sepsis	12 (8.63)	1 (0.72)	11 (7.92)	53740815.68 (0.00 - Inf)	**.989**
OF	42 (30.22)	15 (10.79)	27 (19.42)	5.34 (2.35–12.15)	**<.001**
MOF	23 (16.55)	6 (4.32)	17 (12.23)	12.86 (2.85–57.95)	**<.001**
POF	28 (20.14)	7 (5.04)	21 (15.11)	6.55 (2.31–18.57)	**<.001**
Type of OF, n (%)					
Respiratory failure	26 (18.71)	8 (5.76)	18 (12.95)	5.78 (1.83–18.23)	**.003**
Cardiovascular failure	16 (11.51)	4 (2.88)	12 (8.63)	15.49 (1.95–122.78)	**.009**
Renal failure	16 (11.51)	5 (3.60)	11 (7.92)	6.63 (1.82–24.12)	**.004**
Liver failure	10 (7.19)	2 (1.44)	8 (5.76)	55695027.07 (0.00 - Inf)	.988
Gastrointestinal failure	9 (6.47)	4 (2.88)	5 (3.60)	2.26 (0.64–9.44)	.263
Coagulation failure	1 (0.72)	0 (0.00)	1 (0.72)	2310741.79 (0.00 - Inf)	.987
Extrapancreatic infection, n (%)	19 (13.67)	5 (3.60)	14 (10.07)	4.20 (1.44–12.23)	**.008**
ICU stay, n (%)	38 (27.34)	12 (8.63)	26 (18.71)	4.72 (2.02–11.08)	**<.001**
APACHE II score	6.71 ± 4.88	5.63 ± 3.59	8.21 ± 5.97	1.13 (1.04–1.24)	**.004**
MCTSI score	6.62 ± 1.59	6.30 ± 1.38	7.07 ± 1.77	1.20 (0.96–1.50)	.112
WBC* (×10^9^/L)	13.07 ± 4.83	12.65 ± 4.83	13.66 ± 4.82	1.09 (1.02–1.18)	**.018**
CRP* (mg/L)	161.01 (56.80–263.68)	135.37 (41.06–232.20)	198.55 (121.19–283.72)	1.01 (1.01–1.01)	**.030**
PCT* (mg/dL)	0.76 (0.24–3.22)	0.53 (0.15–1.52)	2.05 (0.56–8.328)	1.17 (1.05–1.30)	**.006**

Bold values indicate statistical significance (*P* < .05).

95% CI = confidence interval, ACS = abdominal compartment syndrome, APACHE II score = acute physiology and chronic health evaluation II, ARDS = acute respiratory distress syndrome, CRP = C-reactive protein, ICU = intensive care unit, MCTSI = Modified Computed Tomography Severity Index, MOF = multiple organ failure, organ failure ≥2 was defined as MOF, OF = organ failure, OR = odds ratio, PCT = procalcitonin, POF = persistent organ failure, organ failure ≥48 hours was defined as POF, SIRS = systemic inflammatory response syndrome, WBC = white blood cell.

* Consecutive maximum data within 3 days of admission

**Figure 2.**
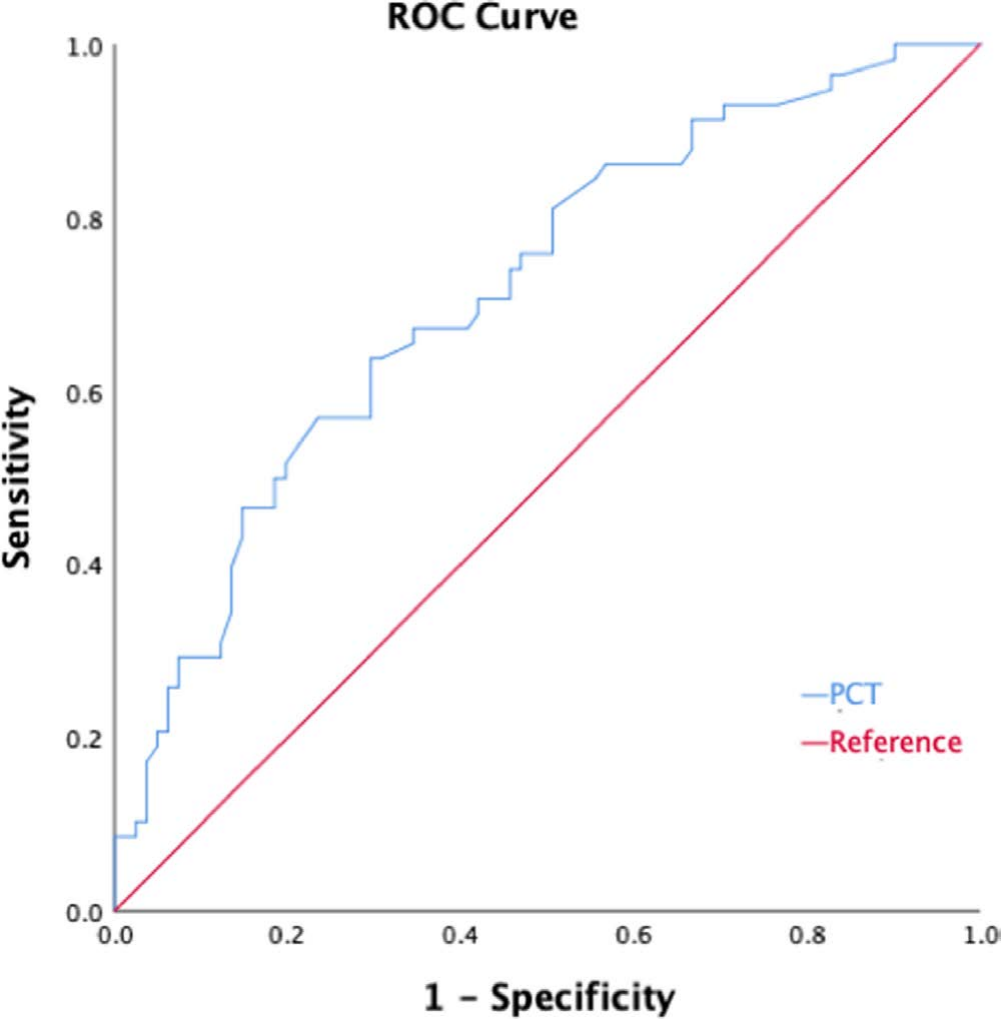
Receiver operating characteristic curve for PCT levels related to the development of INPs. INP = infected necrotizing pancreatitis, PCT = procalcitonin.

The results of the multivariate analysis obtained from the stepwise regression of the above univariate factors are presented in Table 4. According to our multivariate analysis, POF (*P* = .029, OR = 3.32), the pancreatic necrosis area (30–50%, *P* = .029, OR = 3.73; >50%, *P* = .005, OR = 5.18), and the PCT account (*P* = .049, OR = 1.08) were significantly associated with INP. Furthermore, the ROC curve (Fig. 2) showed that the cutoff value of 1.01 mg/dL for PCT was the best predictor of infection (Table 5).

**Table 4:** Multivariate analysis of factors contributing to infection.

	OR (95%CI)	*P*-value
POF	3.32 (1.13–9.75)	.029
Pancreatic necrosis area		
30–50% >50%	3.73 (1.15–12.14) 5.18 (1.65–16.22)	.029 .005
PCT	1.08 (1.01–1.16)	.049

95% CI = confidence interval, OR = odds ratio, PCT = procalcitonin, POF = persistent organ failure, defined as POF.

**Table 5:** The performance of PCT associated with the development of INP.

Variable	AUC (95% CI)	Cutoff	Sensitivity	Specificity
PCT	0.716 (0.630–0.801)	1.01	0.638	0.704

AUC = area under the curve, 95% CI = confidence interval, INP = infected necrotizing pancreatitis, PCT = procalcitonin.

**Figure 3.**
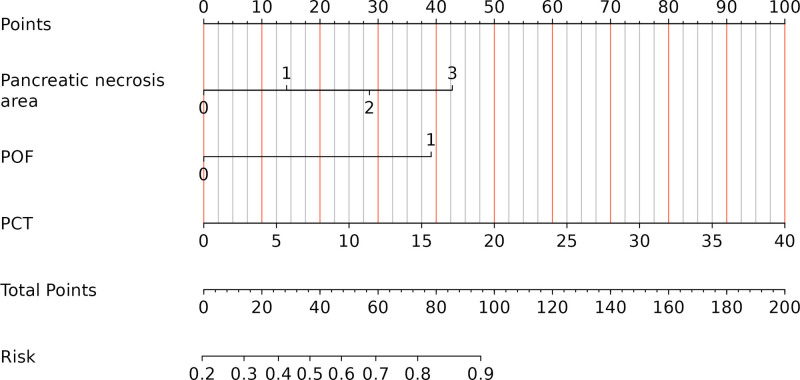
Nomogram for predicting the risk of INP in patients with early MSAP and SAP onset. (A) Training group; (B) validation group. INP = infected necrotizing pancreatitis, MSAP = moderate severe acute pancreatitis, SAP = severe acute pancreatitis.

**Figure 4.**
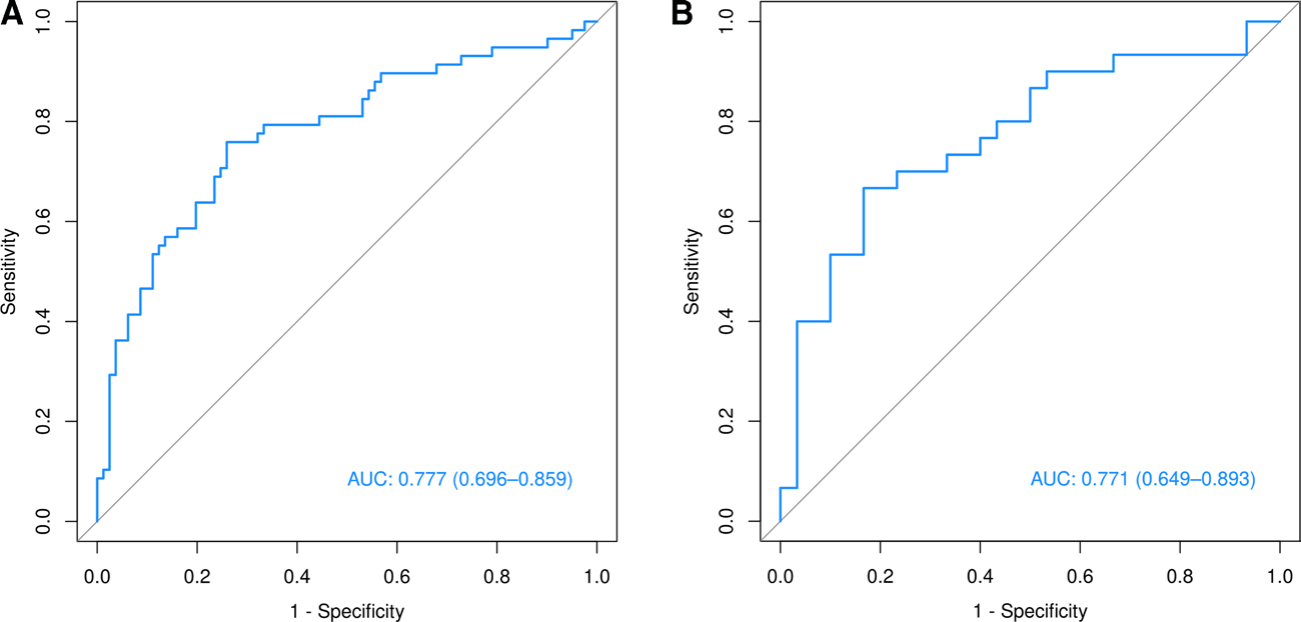
ROC curves of the predictive model. (A) Training group; (B) validation group. ROC = receiver operating characteristic.

**Figure 5.**
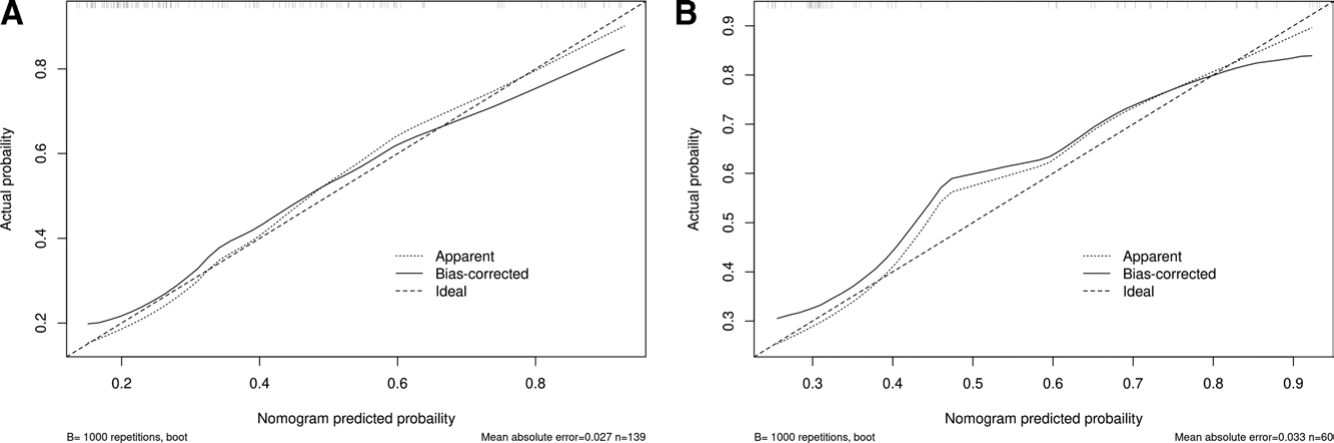
Calibration curves of the predictive models. (A) Training group; (B) validation group.

**Figure 6.**
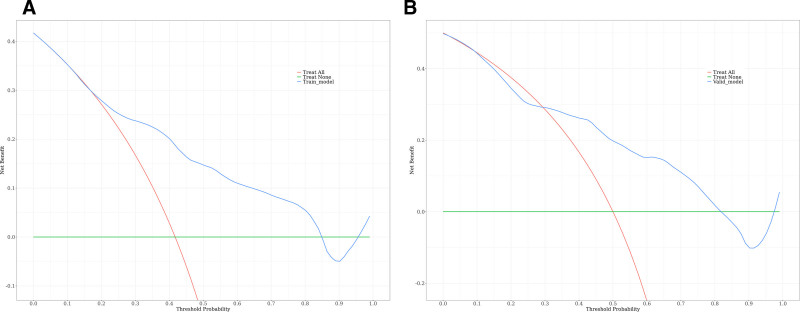
Decision curve analysis of the predictive model.

Based on the predictors selected in the regression analysis, we constructed a nomogram (Fig. 3) for the clinical prediction model and evaluated the discriminatory ability of the model using ROC curves and area under the curve (AUC) (Fig. 4). The ROC curves showed AUCs of 0.777 (0.696–0.859) and 0.771 (0.649–0.893) for the training and validation groups, respectively, suggesting that the model has good discriminating ability. Moreover, the calibration curves (Fig. 5 and Table 6) of the training and validation sets showed that the model’s prediction results fit the actual results well. Moreover, DCA (Fig. 6) showed that the net benefit of the predictive model increased significantly when the threshold was >30%. The nomogram depicted here serves as a predictive tool for assessing the risk of infection in the pancreas and its surrounding tissues in patients with AP. To utilize the nomogram, clinicians first determine individual points for the following clinical indicators: the extent of pancreatic necrosis, the presence of POF, and the patient’s PCT account. These points are then summed to calculate a total score, which is located on the total points row. A vertical line is drawn downward from this total score to the risk row to ascertain the patient’s probability of developing an infection. This probability is represented as a decimal between 0 and 1, with, for example, a value of 0.7 suggesting a 70% chance of infection.

**Table 6:** Hosmer and Lemeshow goodness of fit (GOF) test.

	X-squared	*P*-value
Train group	3.0861	.93
Valid group	13.773	.10

### 3.1. Management and outcomes

Overall, a total of 88 patients eventually progressed to infected necrotizing pancreatitis; 5 (5.68%) patients died, while the remaining patients recovered after conservative treatment, percutaneous catheter drainage, or surgery. Of the patients who developed pancreatic or peripancreatic infections, 71 (80.68%) had infections that occurred in the later stages of AP. All patients with INP underwent percutaneous fine-needle aspiration drainage on admission, with a mean time to first drainage of 5.76 ± 5.16 days after the onset of AP, and the mean number of percutaneous fine-needle aspirations per patient was 2.70 ± 1.26. A total of 61 (69.32%) of patients with INP were given minimally invasive pancreatic debridement, with a mean of 4.01 ± 3.79 procedures. We improved the treatment process according to the step-up approach^[8,9]^ and the actual conditions of the local hospital, including early drainage. After necrosectomy, we performed reperitoneal drainage of the areas with ineffective drainage, and the operative approach was based on the location of infection.

## 4. Discussion

Necrotizing pancreatitis is one of the subtypes of AP, and INP is a pancreatic or peripancreatic infection secondary to necrotizing pancreatitis.^[10]^ Patients with INP may face a complicated and prolonged course of the disease, with an associated mortality rate of 20% to 30%.^[11]^ Therefore, early identification of risk factors that may contribute to INP as well as appropriate interventions are particularly important.

A large number of studies have been conducted to evaluate the ability of the AP severity and mortality rates, while few studies have been conducted to predict infections. Because of early prophylactic antibiotic use, bacterial cultures of many patients may yield false-positive results, increasing the difficulty of diagnosing infections. Existing studies have shown that biomarkers such as PCT,^[12]^ BUN,^[13]^ the absolute lymphocyte count,^[14,15]^ and IL-6^[16]^ may be useful in predicting IPN development. In addition, several scoring systems for determining the severity of pancreatitis, such as the APACHE II score, BISAP score, and Ranson score, have been suggested for predicting the development of INP.^[5,17]^ Nonetheless, these systems are more frequently utilized in the assessment of AP severity, as opposed to being designed for the specific purpose of predicting infectious necrosis. In contrast, the present model focuses exclusively on predicting INP with the inclusion of infection-specific variables such as PCT.

In this study, 4 risk factors associated with INPs, POF, pancreatic necrosis area = 30% to 50%, pancreatic necrosis area > 50% and PCT account > 1.01 mg/dL, were screened by univariate and multivariate logistic regression analyses. A nomogram for determining the risk of INP in patients with MSAP and SAP was constructed and validated based on these independent risk factors. According to the prediction model, the AUC was 0.777 (0.696–0.859) for the training cohort and 0.771 (0.649–0.893) for the validation cohort, indicating that the model has a high prediction capacity. Moreover, the calibration curves of both the training and validation sets showed good agreement between the actual diagnosis and the predicted diagnosis. In addition, the DCA curve also showed that the model had good clinical validity. Notably, all variables included in the model are routinely collected in the early stages of hospitalization, making the model highly feasible for bedside application. This practical advantage allows clinicians to perform early risk stratification, facilitating prompt intervention and potentially improving patient outcomes. In addition, the nomogram can be easily integrated into electronic health record systems, providing a convenient tool to support clinical decision-making in real-time.

During the disease progression of SAP, patients presenting with POF often develop an immunosuppressed state that may increase susceptibility to infection, causing infected necrosis of the pancreas and peripancreas, leading to death.^[18,19]^ Several previous studies^[20,21]^ have shown that approximately 75% of patients with POF develop INP, which is similar to the findings of our center: 31 out of 40 (77.5%) patients with POF develop INP at a late stage.

In our study, pancreatic necrosis > 30% was also an independent risk factor for INP, and several studies have shown that the incidence of pancreatic necrosis is strongly correlated with complications and mortality in AP patients.^[22,23]^ Moreover, Besselink MG et al showed that patients with extensive pancreatic parenchymal necrosis were at increased risk of pancreatic infections.^[24]^ However, the pathophysiological mechanism by which infection occurs in necrotizing pancreatitis is still unclear.

PCT, a commonly used clinical test, has been confirmed to be a biomarker of infection by several studies.^[25,26]^ However, in AP, PCT has been found to be superior for predicting INP and the severity of AP.^[27–30]^ In our study, as an independent risk factor for INP, a maximum PCT > 1.01 mg/dL in the first 3 days after admission was more accurately predicted. Previous studies have shown that a PCT > 0.5 mg/dL seems to accurately predict the severity of SAP.^[12]^ Heterogeneity between patients may cause differences in the PCT cutoff values between our study and others, and our study only statistically analyzed factors that may lead to infection rather than analyzing severity, mortality, or other factors that could be another possible reason for the difference. Elevated serum PCT is closely associated with the host’s inflammatory response to microbial infection.^[26]^ In addition, because the serum PCT concentration decreases as clinical symptoms improve, the PCT concentration is also used as a guide for antibiotic therapy.^[28,31–33]^

In our study, MSAP and SAP secondary to pancreatic infection were present in 44.22% of patients, which is slightly greater than that in previous studies,^[1,2,10]^ but the mortality rate of patients with INP was lower at 5.68%.^[1,8,34]^ This may be due to the application of a modified surgical management system in our center; however, this finding still needs to be corroborated by a large number of prospective studies. Moreover, to avoid the influence of clinical intervention on the variables, we excluded patients who had already been treated in other hospitals, in addition to the heterogeneity of the patients, which could also lead to differences in the results.

This study has several limitations. First, as this was a retrospective study, patients with incomplete clinical data and who attended outside hospitals before admission were excluded. This may introduce a potential selection bias, but this exclusion criterion is intended to minimize the confounding effects of prior therapeutic interventions and to ensure data integrity, though it may limit the representativeness of the sample. Therefore, further validation of the generalisability of the model to real-world clinical situations, particularly in patients referred from other institutions, is required in multicentre prospective cohorts. Furthermore, it is evident that the present study did not directly compare our model with existing ones, such as APACHE II and BISAP. It is therefore recommended that future studies explore the question of whether our model is superior to general severity scores in this specific clinical setting. Such comparative analyses would assist in further determining its utility and unique value. In summary, as this was a single-center study with a limited sample size, the general applicability of the findings may be limited; thus, a larger sample size and more prospective studies are needed for further validation.

In conclusion, we developed a predictive model containing 3 predictive variables, POF, the pancreatic necrosis area and PCT, and the ROC curves, calibration curves and DCA of the training and validation sets showed that the model had good predictive performance. When patients present with these clinical signs, we must be alert to the occurrence of INP, and intervention should be taken at the optimal time to reduce mortality and complications.

## Author contributions

**Conceptualization:** Yu Zhang.

**Data curation:** Shuaiyong Wen.

**Formal analysis:** Yu Zhang, Guijie Zhao.

**Supervision:** Yunfeng Cui.

**Writing – original draft:** Yu Zhang.

**Writing – review & editing:** Yunfeng Cui.
